# New Variants of Tomato Thymidine Kinase 1 Selected for Increased Sensitivity of *E. coli* KY895 towards Azidothymidine

**DOI:** 10.3390/cancers7020819

**Published:** 2015-06-08

**Authors:** Louise Slot Christiansen, Louise Egeblad, Birgitte Munch-Petersen, Jure Piškur, Wolfgang Knecht

**Affiliations:** 1 Department of Biology, Lund University, Lund 22362, Sweden; E-Mail: jure.piskur@biol.lu.se; 2 Lund Protein Production Platform, Lund University, Lund 22362, Sweden; E-Mail: Louise.Egeblad@biol.lu.se; 3 Department of Science, Systems and Models, Roskilde University, Roskilde 4000, Denmark; E-Mail: bmp@ruc.dk

**Keywords:** nucleosides, protein engineering, mutagenesis, suicide gene therapy, deoxynucleosides, deoxynucleotides, deoxynucleoside kinases, nucleoside analog, azidothymidine, prodrug activation

## Abstract

Nucleoside analogues (NA) are prodrugs that are phosphorylated by deoxyribonucleoside kinases (dNKs) as the first step towards a compound toxic to the cell. During the last 20 years, research around dNKs has gone into new organisms other than mammals and viruses. Newly discovered dNKs have been tested as enzymes for suicide gene therapy. The tomato thymidine kinase 1 (ToTK1) is a dNK that has been selected for its *in vitro* kinetic properties and then successfully been tested *in vivo* for the treatment of malignant glioma. We present the selection of two improved variants of ToTK1 generated by random protein engineering for suicide gene therapy with the NA azidothymidine (AZT). We describe their selection, recombinant production and a subsequent kinetic and biochemical characterization. Their improved performance in killing of *E. coli* KY895 is accompanied by an increase in specificity for the NA AZT over the natural substrate thymidine as well as a decrease in inhibition by dTTP, the end product of the nucleoside salvage pathway for thymidine. The understanding of the enzymatic properties improving the variants efficacy is instrumental to further develop dNKs for use in suicide gene therapy.

## 1. Introduction

Deoxyribonucleoside triphosphates (dNTP) for DNA synthesis are provided via *de novo* and salvage pathways. In the human salvage pathway deoxyribonucleosides (dN) are salvaged by four deoxyribonucleoside kinases (dNKs). Furthermore, dNKs are able to phosphorylate nucleoside analogs (NA) that are used for the treatment of viral or cancer diseases and this reaction is usually the rate-limiting step [1]. The diversity of non-viral dNKs and their practical use has been reviewed very recently [2].

Though all non-viral dNKs catalyze the same basic reaction, the transfer of a phosphate group to a deoxyribonucleoside, they can be divided into two structurally different groups: the thymidine kinase 1 (TK1)-like group and the non-TK1-like group [3,4]. The non-TK1-like group has broad substrate specificity and includes the human kinases deoxycytidine kinase (dCK), deoxyguanosine kinase (dGK) and thymidine kinase 2 (TK2), while the TK1-like group has a much more limited substrate specificity, phosphorylating only thymidine (dThd) and deoxyuridine (dUrd) [4].

TK1 is a cytosolic enzyme and during the cell cycle its activity is highly regulated via several mechanisms. The structure-function relationship of human TK1 (HsTK1) is still relatively poorly understood even if HsTK1 has been crystallized [5,6,7]. Each TK1 subunit contains two domains, one α/β domain that is similar to ATPase domains of members of the RecA structural family and is present in several helicases and DNA repair proteins. The other domain is a small domain containing a structural zinc and a unique lassolike loop that covers the deoxyribonucleoside site. The deoxyribose and the base bind in a cleft between the α/β domain and the lasso domain while the triphosphate binds to the α/β domain. The substrate binds by hydrogen bonds only to the main chain atoms of the enzyme and not to the side chains. For this reason it is not obvious to decide which amino acid residues can be substituted to change the substrate specificity [7]. Mutational studies of HsTK1 suggest that Val106 is involved in the quaternary structure of the enzyme [8], but this is not confirmed from the structural analysis [7].

In 2010 the first plant dNK, the TK1 from tomato (ToTK1) was described. ToTK1 phosphorylates the nucleoside analogue 3-azido-2,3-dideoxythymidine (azidothymidine, AZT), a drug originally used to treat HIV patients, equally well as its natural substrate dThd [9]. In addition, it can catalyze the phosphorylation of dThd monophosphate (TMP) and AZT monophosphate (AZT-MP) to their diphosphate compounds, a property not previously reported in other characterized dNKs [9]. In addition ToTK1 in combination with AZT is an interesting potential suicide gene for anti-cancer gene therapy [9,10]. The first dNK used in suicide gene therapy was a viral thymidine kinase from herpes simplex virus (HSV-TK) used in combination with ganciclovir (GCV) as prodrug. This system can therefore be regarded as the prototype model for gene-directed enzyme prodrug therapy [11,12]. This enzyme prodrug combination has been used in phase I/II clinical trials [13,14,15,16] and even a few phase III trials [17,18]. However, lipophobicity of GCV and low enzymatic activity of HSV-TK reduce the treatment efficacy, therefore the search for other enzyme—prodrug combinations is of interest.

The use of non-viral dNKs in suicide gene therapy has also been reviewed recently [2]. Among the three non-viral dNKs that have been tested in *in vivo* animal models so far, we find enzymes from the non-TK1-like group, the ultrafast multisubstrate dNK from *Drosophila melanogaster* (DmdNK) and the human dCK (HsdCK) [19,20,21,22,23,24,25,26,27]. The third dNK is the above described ToTK1 and belongs to a different structural class, the TK1-like family of dNKs. Both enzymes belonging to the non-TK1-like family: DmdNK and HsdCK have been targets for directed evolution and knowledge based protein engineering with the aim to improve performance and optimizing enzyme prodrug combinations. Mutants of DmdNK have been created by directed evolution [28,29], site directed mutagenesis [20,21] or C-terminal truncations have been made [20,30]. In several animal tumor models, wt HsdCK with gemcitabine and a mutated HsdCK with BVDU were tested and showed positive effects on treatment of the tumors [25,26,27].

To the best of our knowledge, no attempts to improve the performance of a dNK belonging the TK1-like group of dNKs for suicide gene-therapy has been reported, yet. ToTK1 is here an obvious candidate in combination with AZT. AZT is in use for many years now and has a well-known and safe profile [31]. When targeting malignant glioma, AZT has been used in combination with ToTK1 in animal models [9,10] and it could be seen as a potential benefit for patients that AZT crosses the blood-brain barrier more readily than GCV [32]. ToTK1 has therefore been proposed as a treatment alternative for malignant glioma as compared to the HSV-TK/GCV system [10].

In this study we have explored if it is possible to improve the catalytic efficacy for AZT over dThd for ToTK1 by random protein engineering and characterize changes in ToTK1 that improves its efficiency as suicide gene in combination with AZT. Our study with random mutagenesis and deletions of ToTK1 provides a better understanding of the strict substrate specificity of TK1 enzymes. We identified several amino acid residues and a protein domain, which play a crucial role in the structure-function relationship related to better performance as suicide gene in our screening system with AZT. The two ToTK1 variants characterized in detail showed that improved performance in cell killing of *E. coli* KY895 is accompanied by an increase in specificity for the NA AZT over the natural substrate dThd as well as a decrease in inhibition of the kinase activity by dTTP, the end product of the nucleoside salvage pathway for dThd. Our results show possibilities and molecular mechanisms important to create new variants of TK1s with increased NA efficacy that can be tested in suicide gene therapy.

## 2. Results and Discussion

### 2.1. Generation and Screening of ToTK1 Mutants

**Figure 1:**
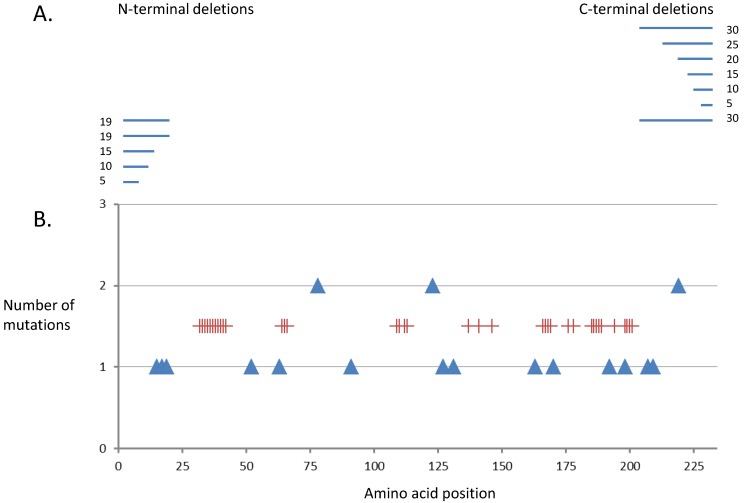
Schematic representation of ToTK1. (**A**) shows the N-and C-terminal deletions made. (**B**) shows number and position of the mutations found in the eight ToTK1 variants selected from the error prone PCR approach. The red crosses indicate the positions of highly conserved amino acids in the TK1-like group of dNKs derived from a multiple alignment of 10 sequences of TK1-like kinases covering plants, amoeba, invertebrates and vertebrates.

Two main approaches were used to create variants of ToTK1. As previously done for DmdNK random mutagenesis was done by error prone PCR using dNTP analogs [28,29]. In parallel, N- and C-terminal truncations were done. C-terminal truncation had been shown for DmdNK to stabilize the enzyme [30] and DmdNK mutated at the very C-terminus is also used in suicide gene therapy testing [20]. In total 11 different N- and C-terminal deletions were made (Table 1, Figure 1) and tested in KY895 cells on screening plates with and without AZT.

**Table 1:** N- and C-terminal deletions of ToTK1 and how they sensitize transformed *E. coli* KY895 cells to AZT. The MIC were from 3 experiments. pGEX-2T stands for cells transformed with the empty expression plasmid. If no concentration range is given, all 3 experiments showed the same MIC.

Deletion	MIC (nM)
pGEX-2T	>31600
wt ToTK1	31.6
ΔN5	10–31.6
ΔN10	10–31.6
ΔN15	10–31.6
ΔN19	10–31.6
ΔC5	31.6
ΔC10	10
ΔC15	10
ΔC20	5–10
ΔC25	5
ΔC30	10
ΔN19/C30	10

From the error prone PCR, a total of 8000 colonies were screened. Eight clones, corresponding to 0.1% of all colonies screened, were identified with an increased sensitivity to AZT (Table 2).

**Table 2:** Mutations found in ToTK1 that sensitize transformed *E. coli* KY895 cells to AZT and their MIC values. The MIC were from three experiments. pGEX-2T stands for cells transformed with the empty expression plasmid. If no concentration range is given, all 3 experiments showed the same MIC.

Amino acid position	15	17	19	52	63	78	91	123	127	131	163	170	192	198	207	209	219	MIC (nM)
**pGEX-2T**																		**>31,600**
**wt ToTK1**	**N**	**S**	**N**	**D**	**K**	**T**	**I**	**N**	**F**	**I**	**T**	**N**	**D**	**Q**	**S**	**N**	**H**	**31.6**
**ToM3**		**W**	**S**									**D**	**N**				**R**	**10**
**ToM4**						**A**		**S**										**10**
**ToM6**	**D**																	**10**
**ToM8**							**T**							**R**				**10**
**ToM5**					**R**			**H**			**A**						**R**	**10**
**ToM1**																**G**		**10**
**ToM7**															**P**			**10**
**ToM2**				**N**		**A**			**S**	**T**								**10**

### 2.2. ToTK1 Mutants

A best lethal dose or minimum inhibitory concentration (MIC) concentration of 5 nM AZT was found for a 25 amino acid deletion at the C-terminus of ToTK1 (Table 1). In general, truncating the N- and C-terminus of ToTK1 had a positive effect with regard to increasing the sensitivity of KY895 towards AZT as compared to wt ToTK1. The observed increase in sensitivity, approximately 6-fold, seems small as compared to improvements achieved in other studies, e.g., with DmdNK [28,29]. However, to the best of our knowledge 5 nM of a NA is the lowest MIC achieved so far in this kind of screening setting.

The eight clones derived from the screening of the error prone PCR generated library of mutants achieved a MIC of 10 nM (Table 2, Figure 1). The mutations were randomly distributed and most of them were found only once among the eight mutants and not in any conserved position. Apart from ToM1, ToM6 and ToM7, all of the mutants contained more than one amino acid substitution. However, based on sequence alignment of ToTK1 to human HsTK1, none of the mutations except for the D192N are close to the active site and it is not obvious what their impact on structure function relationship might be and therefore to what extent they contribute to the decreased MIC value. The amino acid substitutions T78A and H219R were identified twice. Furthermore N123 was also identified in two mutants as N123S and N123H. The above mentioned substitutions were identified in mutants that also contained other substitutions. To test if and to what degree the T78A, H219R and N123S/H where responsible for the increased sensitivity that ToTK1 mutants transfer to KY895, these substitutions where introduced with site-directed mutagenesis in ToTK1. However, when tested in our screening system, none of these ToTK1 mutants showed the same decrease in MIC, as when these amino acid substitutions were present in the random mutants in combination with other substitutions [33]. In conclusion the observed better performance in cell killing down to an MIC of 10 nM cannot be solely correlated to a single amino acid exchange in ToTK1.

### 2.3. Characterization of ToTK1 Mutants

Previous studies done with mutants of DmdNK derived by directed evolution demonstrated that the molecular mechanisms behind the improved properties of the mutant enzymes are caused by altered substrate specificity and modified feedback inhibition [28,29]. The structural basis for these changes could be explained by crystallographic studies of a DmdNK mutant selected for increased sensitivity to AZT (MIC = 316 nM) in complex with substrate and feedback inhibitor [34]. However, ToTK1 is a member of the TK1-like group of dNKs with a completely different structure than dNKs as DmdNK of the non-TK1-like group [3,4].

We therefore attempted to characterize the molecular mechanisms for the increased sensitivity towards AZT that ToTK1 mutants transfer to KY895. For this we selected the best mutant ToTK1ΔC25 as a representative of the truncated enzymes and one representative of the mutants derived from error prone PCR. Here, we chose ToTK1M4, as both identified mutations were located quite central in the sequence of the 234 amino acid long ToTK1 (not covered by the truncations) and mutations at both positions were also found in other variants (Table 2).

#### 2.3.1. Expression and Purification of wt ToTK1 and Two ToTK1 Mutants

**Figure 2:**
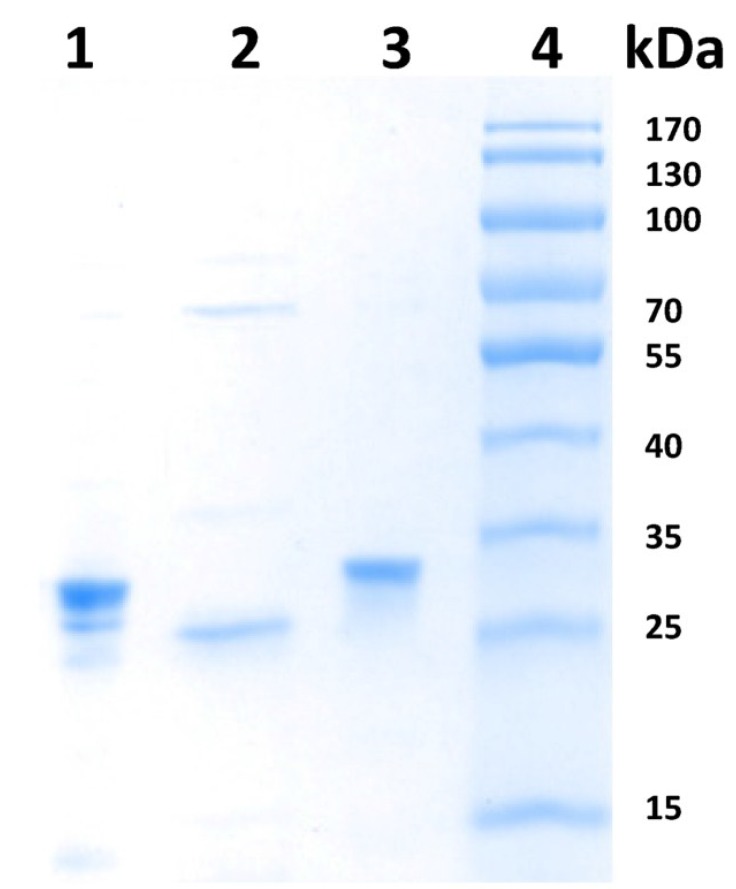
Coomassie stained SDS-PAGE of purified wt ToTK1 and mutants. 2 µg of protein was applied per lane. 1: wt ToTK1; 2: ToTK1ΔC25; 3: ToTK1M4; 4: Marker.

The expression and purification of the thrombin-cleaved recombinant enzymes was done as described earlier for dNKs expressed from pGEX-2T [9,29,35]. Figure 2 presents a Coomassie stained SDS-PAGE with preparations used in this study. Differences in the properties of the enzymes with respect to expression and purification were observed for the three enzymes. While wt ToTK1 seemed to be prone to degradation, as indicated by smaller bands, ToTK1ΔC25 carried a higher MW contamination into the final prep. In both mutant cases, the lack of smaller bands indicates a better stability towards degradation than for wt ToTK1. With the imaging software image lab V.4 (Biorad), the preparations were judged to be 80% for wt ToTK1 and ToTK1ΔC25 and apparent homogenous for ToTK1M4. Calculation of the kinetic data as the V_max_ values in Table 3 were adjusted for the grade of homogeneity.

**Table 3:** Kinetic parameters of wt ToTK1 and mutant ToTK1s. The kinetic parameters and the increase in specificity (-fold) of the mutants when compared to wild-type were calculated as outlined in the experimental section. K_m_ and V_max_ values are presented as average and SD from three experiments.

Enzyme	Mutation	K_m_ (μM)	V_max_ (nmol·min^−1^·mg^−1^)	K_cat_ (s^−1^)	K_cat_/K_m_ (M^−1^s^−1^)	Increase in specificity (-fold)
**dThd**						
**Wt ToTK1**	wt	0.4 ± 0.06	3660 ± 130	1.58	3.95 × 10^6^	
**ToTK1M4**	T78A; N123S	1.3 ± 0.36	1600 ± 603	0.69	5.31 × 10^5^	
**ToTK1ΔC25**	deltaC25	1.6 ± 0.1	5170 ± 44	1.99	1.24 × 10^6^	
**dUrd**						
**Wt ToTK1**	wt	17 ± 0.5	3400 ± 215	1.47	8.65 × 10^4^	
**ToTK1M4**	T78A; N123S	15 ± 3.3	1900 ± 289	0.82	5.47 × 10^4^	
**ToTK1ΔC25**	deltaC25	27 ± 3.3	4000 ± 193	1.54	5.7 × 10^4^	
**AZT**						
**Wt ToTK1**	wt	1.2 ± 0.04	3200 ± 24	1.38	1.15 × 10^6^	
**ToTK1M4**	T78A; N123S	1.1 ± 0.1	740 ± 122	0.32	2.91 × 10^5^	1.5
**ToTK1ΔC25**	deltaC25	2 ± 0.06	2470 ± 190	0.95	4.75 × 10^5^	1.2

#### 2.3.2. Substrate Specificities of wt ToTK1 and Two ToTK1 Mutants

The kinetic parameters of wt ToTK1 and the two mutants were determined for the two natural occurring substrates dThd and dUrd as well as for the NA AZT (Table 3).

The wildtype enzyme exhibited the highest catalytic efficiency (k_cat/_K_m_) with dThd (3.95 × 10^6^ M^−1^s^−1^), which was 3.4 times higher than with AZT (1.15 × 10^6^ M^−1^s^−1^) and 46 times higher than with dUrd (8.65 × 10^4^ M^−1^s^−1^). The lowest K_m_ value was obtained with dThd *i.e.*, 0.4 µM, while it was 1.2 µM and 17 µM for AZT and dUrd, respectively. The determined V_max_ values were in the same range.

The ToTK1M4 showed lower catalytic efficiencies for all substrates than the wildtype. The highest catalytic efficiency was with dThd, 5.31 × 10^5^ M^−1^s^−1^, which was 1.8 times higher than with AZT (2.91 × 10^5^ M^−1^s^−1^) and 9.7 times higher than with dUrd (5.47 × 10^4^ M^−1^s^−1^). The lower efficiencies were mainly due to lower V_max_ values of 1.6 U/mg, 0.74 U/mg and 1.9 U/mg for dThd, AZT and dUrd, respectively (1 U equals 1000 nmol/min). The lowest K_m_ value was obtained with AZT, 1.1 µM, which was similar to the wildtype value. However, the K_m_ for dThd was increased three times (1.3 µM) compared to wildtype. The K_m_ with dUrd was 15 µM.

Like the two other enzymes, the ToTK1ΔC25 exhibited the highest catalytic efficiency 1.24 × 10^6^ M^−1^s^−1^ with dThd, which was 2.6 times higher than with AZT (4.75 × 10^5^ M^−1^s^−1^) and 22 times higher than dUrd (5.7 × 10^4^ M^−1^s^−1^). The K_m_ value for dThd (1.6 µM) was 4 times higher than the wildtype, whereas the K_m_ for AZT (2 µM) was less affected and only 1.8 times higher. However the ToTK1ΔC25 exhibited a much higher V_max_ with dThd of 5.2 U/mg than the wildtype, while the V_max_ for AZT (2.5 U/mg) was lower than for the wildtype.

The increase in specificity for AZT was calculated as previously described for NA using the equation (k_cat_/K_m_(NA))/(k_cat_/K_m_(dUrd) + k_cat_/K_m_(dThd) + k_cat_/K_m_(NA)) for each enzyme to determine its specificity and then comparing the resulting specificities [28,29].

We conclude that as previously found for other dNK mutants from the non-TK1-like group, changed substrate specificities are found in the ToTK1 mutants when compared to wt ToTK1. However, the increase in specificity for AZT is slightly lower in the ΔC25 mutant than in M4 (Table 3), despite the observation that ToTK1ΔC25 transfers a lower MIC to KY895 (Table 1 and Table 2).

#### 2.3.3. Inhibition of wt ToTK1 and Two ToTK1 Mutants by dTTP

Feedback regulation of enzyme activities plays a major role in the control of metabolic pathways. In the case of dNKs of the TK1-like family, dTTP is the end product of the dThd salvage pathway and has to be considered as major feedback inhibitor of the enzyme. ToTK1 has previously been shown to be less prone to dTTP mediated inhibition than human TK1 [36], a feature that has been proposed as advantageous for the use of ToTK1 in suicide gene therapy.

IC_50_ values for the half maximal inhibition of wt ToTK1 and mutant ToTK1s by dTTP were determined at two concentrations of the substrates dThd and the NA AZT. The IC_50_ values deliver an easy readout of the inhibitory effect of dTTP under comparable substrate conditions for all three enzymes (Table 4).

As can be seen from Table 4, at low concentrations of substrate (1 µM), the effective concentration of dTTP needed to achieve half maximal inhibition of the mutants in comparison to the wt ToTK1 increases (with *p*-values of 0.021 and 0.03 for dThd and 0.013 and 0.08 for AZT for ToTK1M4 and ToTK1ΔC25, respectively). At high concentrations of substrate (10 µM) this effect is less pronounced or absent. dThd concentrations have only been determined in extracellular fluids with an average of 0.5 µM in humans and 4.9 µM in general [37]. The expected plasma concentrations of AZT in humans depend on the dosing, however as peak concentrations after single oral dosage, concentrations of 6.5–7.3 µM, were reported [38]. dTTP intracellular is reported with an average concentration of 37 ± 30 µM [37]. This suggests that the mutant ToTK1s developed by us, should have an advantage compared to wt ToTK1 e.g., being less inhibited by dTTP under physiological conditions.

**Table 4:** Inhibition of wt ToTK1 and mutant ToTK1s by dTTP. The IC_50_ values were calculated as outlined in the experimental section. IC_50_ values are presented as average and SD from three experiments. The significance of the difference in IC_50_ with increased substrate concentration was tested by unpaired t-test and the p values are given in brackets.

Enzyme	Substrate (µM)	IC_50_ dTTP (µM)	
		dThd	AZT
Wt ToTK1	10	49.1 ± 1.7 (0.026)	71.7 ± 11.1 (0.01)
	1	25.4 ± 11.7	26.8 ± 12.3
ToTK1M4	10	55.6 ± 5.2 (1)	63.8 ± 12.1 (0.77)
	1	55.6 ± 7.9	61.3 ± 6.9
ToTK1ΔC25	10	65.2 ± 6.0 (0.021)	93.9 ± 21.4 (0.016)
	1	49.3 ± 4.4	43.8 ± 2.8

Though the mutant ToTK1s developed in this study have yet to be tested in cancer cells they would be expected to cause a greater toxicity than the wt enzyme. This partly because they are less inhibited of dTTP than the wt enzyme and should consequently phosphorylate more AZT and partly because both mutant enzymes have increased specificity for AZT compared to the wt enzyme and will more efficiently phosphorylate AZT.

In our bacterial test system, at very low AZT concentration in the low nM range, it is very likely that the decrease in susceptibility towards dTTP inhibition is a major factor for the increased sensitivity for AZT transferred to KY895 cells by ToTK1M4 or ToTK1ΔC25 compared to ToTK1. We can however not account for which factor, the increased substrate specificity (Table 3) or the decreased inhibition by dTTP (Table 4) contributes to what degree to the decreased AZT MIC observed for KY895 transformed with the mutant ToTK1s. We can however conclude that likewise as seen in non-TK1-like dNK mutants evolved in a similar manner [28,29], an increase of specificity for the NA and a decrease in dTTP feedback inhibition is also found in mutants derived from a member of the TK1-like dNK family.

The shift in IC_50_ values at different substrate concentrations can deliver additional mechanistic information about the inhibition type and if the inhibition type is known an inhibition constant for the inhibitor can be calculated [39,40]. The results for ToTK1 and the mutant ToTK1s are summarized in Table 5. The significant increase of dTTP IC_50_ with substrate concentration for wt ToTK1 and ToTK1ΔC25 indicates competitive inhibition of dTTP with respect to dThd and AZT (Table 4). For the mutant ToTK1M4, we found non-competitive inhibition, as the IC_50_ stays the same regardless of substrate concentration (Table 4). The mutations do not only change substrate specificity but also alter the mechanism of inhibition by dTTP in the case of ToTK1M4. A change of inhibition mechanism has been previously reported for a mutant of DmdNK evolved by directed evolution for AZT usage, however in this case from uncompetitive to competitive inhibition type [28].

**Table 5:** Mechanism of wt ToTK1 and mutant ToTK1s inhibition by dTTP. The K_ic_ or K_i_ values were calculated from the IC_50_ values from Table 4 and the K_m_ values from Table 3 as outlined in the experimental section. They are reported as average of all data.

Varied Substrate	dThd		AZT	
Enzyme	K_ic_ or K_i_ (µM) for dTTP	Type of inhibition	K_ic_ or K_i_ (µM) for dTTP	Type of inhibition
Wt ToTK1	4.6	competitive	11.1	competitive
ToTK1M4	55.6	non-competitive	62.6	non-competitive
ToTK1ΔC25	19.4	competitive	22.3	competitive

The feedback inhibition of non-TK1-like kinases has been characterized and is complex [41]. With dThd, dTTP displayed uncompetitive inhibition. Here we can show that with wt ToTK1, we find competitive inhibition of dTTP with respect to dThd or AZT. This seems to be a newly described distinguishing feature between TK1-like and non-TK1-like dNKs. However, to rationalize the changes introduced by the double mutation T78A and N123S in ToTK1M4 with respect to its changed inhibition pattern by dTTP, a determination of the crystal structure of ToTK1 and its mutant would be desirable. It is however likely that dTTP binds in the triphosphate binding site. This can be postulated from the inhibition pattern seen in other dNKs, where dTTP inhibition always includes competition with the phosphate donor at the phosphate donor binding site of the enzyme [41] and in wt ToTK1 this binding would then overlap with the binding of dThd in the deoxyribonucleoside site. Hereby we would explain the competitive pattern seen with this enzyme with respect to dThd. To explain the shift in inhibition pattern, dTTP would still bind in the triphosphate binding site in the mutant and inhibit the enzymatic reaction, however the mutations would cause that its binding does not anymore overlap with the binding of dThd and blocks its binding into the deoxyribonucleoside site. Such a scenario would fit with all dTTP inhibition patterns for non-viral dNKs so far [41].

## 3. Experimental Section

The wt ToTK1 (Genebank accession number: NM_001247784.1) in pGEX-2T (GE Healthcare, Uppsala, Sweden) was previously described [9] and used here as a template for the creation of random mutants and N- or C-terminal deletions.

### 3.1. Random Mutagenesis and Screening

The random ToTK1 mutants were generated using 5 µM mutagenic nucleoside analogues (8-oxo-dGTP and dPTP) in a standard PCR reaction with Taq polymerase. The randomly mutagenized PCR fragments were subcloned into the multiple cloning site of pGEX-2T. The TK-deficient *E. coli* KY895 (*F−, tdk-1, ilv*) [28,29,42] was electrotransformed with the ligation mix and the library was then kept in the transformed cells. Single colonies were picked from the library and transferred to 384-well microtiter plates containing LB-medium with 100 µg/mL ampicillin and 10% glycerol and inoculated overnight. The overnight cultures were replicated to 384 well plates with LB and 100 µg/mL ampicillin and transferred to screening plates (1 × M9, 0.2% glucose, 1 g/L casamino acids, 0.1 mM CaCl_2_, 2 mM MgSO_4_, 5 mg/L thiamine, 100 µg/mL ampicillin) containing various concentrations of AZT, with a 384-pin replicator. Growth of colonies was visually inspected after 16 hours at 37 °C. From clones not growing on AZT-containing plates, but growing on plates without NA, the plasmid was re-isolated from the master plate and retransformed into fresh KY895. The obtained clones were retested to verify the plasmid borne phenotype. The lethal dose or minimum inhibitory concentration (MIC) of AZT was determined as the lowest concentration of the analog still completely inhibiting cell growth.

### 3.2. N- and C-Terminal Deletions of ToTK1

The inserts for subcloning were obtained by PCR. For C-terminal deletions several reverse primers were designed to contain an EcoRI and a stopcodon (TAA) just downstream from the deletion site. For N-terminal deletions forward primers with BamHI sites just upstream the truncated coding sequence were designed and used in combination with a reverse primer containing a EcoRI site and a stop codon. The amplified fragments were subcloned into pGEX-2T BamHI/EcoRI and the inserts sequence was verified with sequencing. MIC values were determined as described above.

### 3.3. Purification of Mutants and Enzyme Assays

Expression and purification of thrombin-cleaved recombinant wt ToTK1 or mutants were done as described earlier for dNKs expressed from pGEX-2T [9,29,35]. Nucleoside kinase activities were determined using ^3^H-labeled nucleoside substrates and assays were carried out as described previously [30]. ^3^H labeled dThd was from Perkin-Elmer (Upplands Väsby, Sweden) and other radioactive labeled nucleosides were from Moravek Biochemicals (Brea, CA, USA).

Kinetic data were evaluated by non-linear regression analysis using the Michaelis-Menten equation v = V_max_*[S]/(K_m_ + [S]) as described earlier [43].

The specificity of each enzyme for a single NA can be described using the equation (k_cat_/K_m_(AZT))/(k_cat_/K_m_(dThd) + k_cat_/K_m_(dUrd) + k_cat_/K_m_(AZT)) [28,29]. A comparison assumes that the concentrations of the nucleosides are similar in the systems compared.

IC_50_ values for the inhibition of enzymatic activity by dTTP were calculated by non-linear regression analysis using the equation Q = V/(1 + ([I]/IC_50_)^n^) as described earlier [39]. Q denotes the assay readout, V denotes the uninhibited velocity of the enzyme, [I] denotes the inhibitor concentration, n denotes the slope and IC_50_ is the inhibitor concentration at V/2. To be able to compare the binding affinity of dTTP between the different enzymes, we converted the IC_50_ into K_ic_ values for competitive inhibitors using the equation K_ic_ = IC_50_/(1 + [S]/K_m_) [39]. [S] denotes the substrate concentration in the assay. For a non-competitive inhibitor, the IC_50_ equals the inhibition constant K_i_ [40]. Unpaired *t*-test was performed using GraphPad (GraphPad Software, Inc., San Diego, CA, USA).

## 4. Conclusions

We present here the generation of ToTK1 mutants that transfer an increased sensitivity towards AZT to *E. coli* KY895. The mutations found are outside highly conserved regions of the enzyme. As in similarly evolved mutants of the non-TK1-like group of dNKs, we demonstrate here, that also mutants derived from a TK1-like dNK show an increase in substrate specificity for the NA selected for and a decrease in dTTP inhibition. This may indicate a general molecular mechanism to improve dNKs for more effective suicide gene prodrug combinations. Nevertheless, the mutant ToTK1s developed in this study have yet to be tested in cancer cells.

However, to fully rationalize the functions of the double mutation T78A and N123S in ToTK1M4, in particular with respect to its changed inhibition pattern by dTTP, or of the deleted last 25 amino acids in ToTK1ΔC25 a determination of the crystal structure of ToTK1 and its mutants would be necessary. Besides random protein engineering as used by us here, the understanding of ToTK1 structure function relationship by structure determination will be instrumental to further developing and improving tomato ToTK1 for use in suicide gene therapy by knowledge based directed mutagenesis.
